# Bibliometric Analysis of Current Status on Bioremediation of Petroleum Contaminated Soils during 2000–2019

**DOI:** 10.3390/ijerph18168859

**Published:** 2021-08-23

**Authors:** Yingjin Song, Ruiyi Li, Guanyi Chen, Beibei Yan, Lei Zhong, Yuxin Wang, Yihang Li, Jinlei Li, Yingxiu Zhang

**Affiliations:** 1 School of Environmental Science and Engineering, Tianjin University, Tianjin 300072, China; yingjin@tju.edu.cn (Y.S.); lruiyi1998@163.com (R.L.); chen@tju.edu.cn (G.C.); yanbeibei@tju.edu.cn (B.Y.); lei.zhong@tju.edu.cn (L.Z.); wangyuxin2020@tju.edu.cn (Y.W.); liyihang_1996@tju.edu.cn (Y.L.); lijl@tju.edu.cn (J.L.); 2 School of Mechanical Engineering, Tianjin University of Commerce, Tianjin 300134, China; 3 China-Australia Centre for Sustainable Urban Development, Tianjin 300350, China

**Keywords:** bioremediation, soil, petroleum contamination, bibliometric analysis, VOSviewer

## Abstract

Petroleum contaminated soils have become a great concern worldwide. Bioremediation has been widely recognized as one of the most promising technologies and has played an important role in solving the issues of petroleum contaminated soils. In this study, a bibliometric analysis using VOSviewer based on Web of Science data was conducted to provide an overview on the field of bioremediation of petroleum contaminated soils. A total of 7575 articles were analyzed on various aspects of the publication characteristics, such as publication output, countries, institutions, journals, highly cited papers, and keywords. An evaluating indicator, *h*-index, was applied to characterize the publications. The pace of publishing in this field increased steadily over last 20 years. China accounted for the most publications (1476), followed by the United States (1032). The United States had the highest *h*-index (86) and also played a central role in the collaboration network among the most productive countries. The Chinese Academy of Sciences was the institution with the largest number of papers (347) and cooperative relations (52). Chemosphere was the most productive journal (360). Our findings indicate that the influence of developing countries has increased over the years, and researchers tend to publish articles in high-quality journals. At present, mainstream research is centered on biostimulation, bioaugmentation, and biosurfactant application. Combined pollution of petroleum hydrocarbons and heavy metals, microbial diversity monitoring, biosurfactant application, and biological combined remediation technology are considered future research hotspots.

## 1. Introduction

Petroleum is composed of complicated mixtures of refractory components, such as n-alkane, aromatics, resins, and asphaltenes [1]. As the main energy in the world today [2], the demand for petroleum has increased dramatically with the continuous accelerating process of industrialization. In the process of exploitation, refining, storage, and transportation, petroleum and its products leak into the environment, causing pollution to farmland, soil, rivers, oceans, and so on, resulting in a series of environmental problems [3,4]. According to Russian scientists, 7% of the world’s crude oil extracted goes into the environment every year [5].

Soil usually is the final destination of many organic or inorganic pollutants. Petroleum hydrocarbons will gradually release into the atmosphere or in water, while they are easy to accumulate in soil [6]. For example, approximately 90% of the world’s polycyclic aromatic hydrocarbons (PAHs) settle in soil [7]. When petroleum enters soil, on the one hand, it changes the physicochemical properties of soil, destroys the gas, liquid, and solid structure, and affects soil fertility and crop productivity [8]. On the other hand, its toxicity inhibits the growth of indigenous microorganisms, impeding the respiration and absorption of nutrients and water by plant roots, leading to malnutrition and even death of plants [9]. What is more, harmful substances in petroleum accumulate in soil for a long time and enter the food chain through agricultural crops, causing serious threats to the health of humans as well as other animals [2]. According to statistics, petroleum entering the ecosystem accounts for over 5 million deaths annually [10]. Petroleum contaminated soils (PCS) have become a great concern worldwide.

At present, there are mainly three remediation technologies for PCS: physical, chemical, and biological remediation. Physical and chemical remediation take a short time and have a rapid onset of action, but they are costly and may cause secondary pollution of soil [11]. Bioremediation is considered the most promising environmental remediation technology because of its low cost, easy operation, small ecological risk, and broad scope of remediation [12,13,14,15]. Bioremediation is an eco-friendly method that uses plants and animals as well as microorganisms to degrade environmental contaminants into less toxic forms [16]. It accelerates the natural biodegradation rate by optimizing the environmental conditions, controlling the limiting factors, and so on [4]. A huge number of experimental results and practical applications have already demonstrated the feasibility of bioremediation for the degradation of pollutants such as petroleum hydrocarbons in soil [13,17,18]. In the circumstances of PCS becoming increasingly serious, it is of great significance to study the bioremediation technology of contaminated soils.

Bibliometrics is a quantitative analysis of publications in a certain scientific field that uses mathematical and statistical methods to clarify the historical progress, research trends, and hot issues in the field to predict the future development and research direction of the particular discipline [19,20,21]. It can analyze the publication year, country, organization, journal distribution, keywords, and other information from a large number of publications in a short time and can visualize the spatial distribution of publication authors, cooperation between countries/organizations, and co-citation of keywords through some auxiliary means [22,23]. Bibliometrics has been widely used in environmental science [24], biology [25], economics [26], and other fields because of its macrocosmic approach, objectivity, and accuracy [27]. In recent years, some studies have analyzed the current situation of petroleum and its derivatives in contaminated soils based on bibliometric analysis. Quintella et al. [28] reviewed the bioremediation technologies of different types of contaminated matrices, bioremediation agents, and contaminants. Mao et al. [6] examined the current status and future trends of contaminated soil remediation from 1996 to 2015. The results indicated that bioremediation is the most significant research direction for treating PCS.

Accordingly, the present study aimed to apply a bibliometric approach using VOSviewer visualization software as a research tool to analyze the relevant literature from the Web of Science (WOS) database in the field of PCS bioremediation over the period 2000–2019 in order to explore the current state of research in this field, reveal the research hot spots and development prospects, and provide references for subsequent investigation.

## 2. Methodology and Data

### Data Collection

The bibliometric analysis of PCS bioremediation was conducted using the Web of Science (WOS) Core Collection database, which is considered the optimal database for bibliometric analysis because of its comprehensive data [29,30]. Terms, Boolean operators, and parentheses were used to create the search: “TS = ((soil*) AND (biodegradation OR (biological degradation) OR bio-degradation OR bioremediation OR bio-remediation OR (biological remediation) OR (microbial remediation) OR (microbial degradation)) AND (oil OR petroleum OR hydrocarbon* OR diesel OR fuel OR benzine))”. The time span was specified as the 2000–2019 period. All data were collected on 2 December 2020 to avoid changes in the number of publications and citations. Data for 2020 were excluded, as this year was incomplete at the time this paper was written. In total, 8670 publications met the selection criteria. These documents were categorized into 4 major types: articles (7575; 87.4%), conference papers (850; 9.8%), reviews (503; 5.8%), and others (69; 0.8%). In this study, only the type of “article” was considered, as the citation data for articles were a more reliable reflection of the research trend. All of the search results were exported in tab-delimited (Windows) format, which included bibliographical information such as titles, authors, journals, institutions, author keywords, publication years, and abstracts for further interpretation and visualization through bibliometric analysis software.

VOSviewer is a widely used bibliometric application software developed by Leiden University to evaluate the current status of research and hot spots in a field that enables visual analysis of the published literature by country, research institution, keyword, etc. [31]. It exhibits unique advantages in graphic presentation, especially in “co-occurrence” network clustering [32]. In essence, VOSviewer automatically processes semantic clustering to identify the relationships between items and generates co-occurrence network maps. In this study, analyses of publications were implemented by Microsoft Excel and VOSviewer. The number of publications (according to year, country, institution, and journal) and the annual variation trend of publications (according to year and major countries), as well as highly cited literature, were analyzed using Microsoft Excel. Analysis of the co-authorship relationships and co-occurrence relationships was implemented by VOSviewer.

## 3. Results and Discussion

### 3.1. Growth Trend of Publications

**Figure 1:**
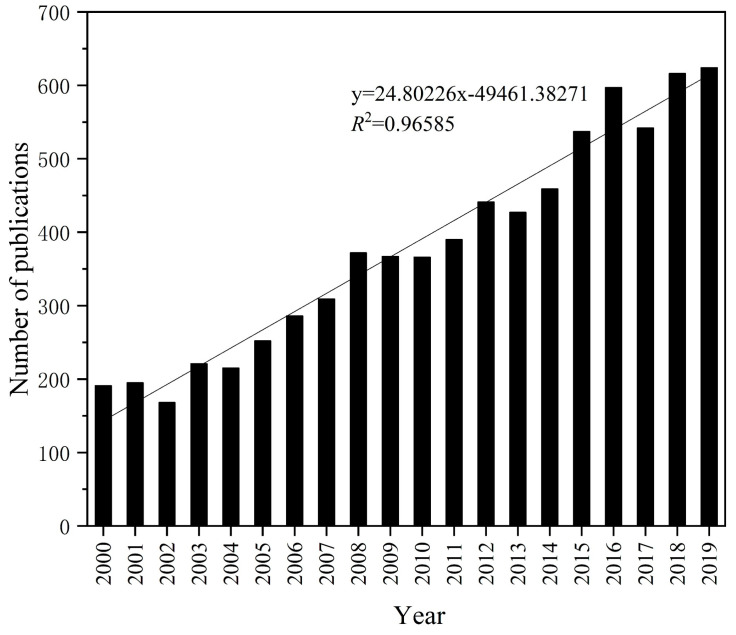
Quantity of annual publications on bioremediation of PCS during 2000–2019.

The annual volume of publications reflects the level and growth of a field to a certain degree [33]. As shown in Figure 1, the number of publications on the bioremediation of PCS in the WOS presented a fluctuating upward scenario, and the relationship between annual volume and year was fitted with a linear regression model. The number of published articles grew from 191 in 2000 to 624 in 2019, with an average annual growth rate of 21.7%, which indicates that the field of PCS bioremediation had gained increasing attention in the last two decades and that the number of scientific research achievements had been increasing. Nonetheless, neither the total number of publications nor the growth rate had greatly improved, which indicates that there was still a large space for development. It can be predicted that the bioremediation of PCS will be a research hotspot for researchers in the coming years.

Characteristics of the annual production are illustrated in Table 1. As the table shows, with a similar increase in the annual number of articles, the number of references cited per article increased significantly over the years. The number of references per article was 30.7 in 2000, compared with 50.8 in 2019—an obvious increase over the course of this 20-year period. In addition, there was an average of 3.1 authors per article in 2000, and this number increased to 4.5 in 2019. The average article length fluctuated slightly, with an overall average of 9.4 pages. The growth in the number of references and authors of publications reflect that development in the field of PCS bioremediation increased steadily during the past 20 years.

### 3.2. Publication Distribution of Countries/Territories

**Figure 2:**
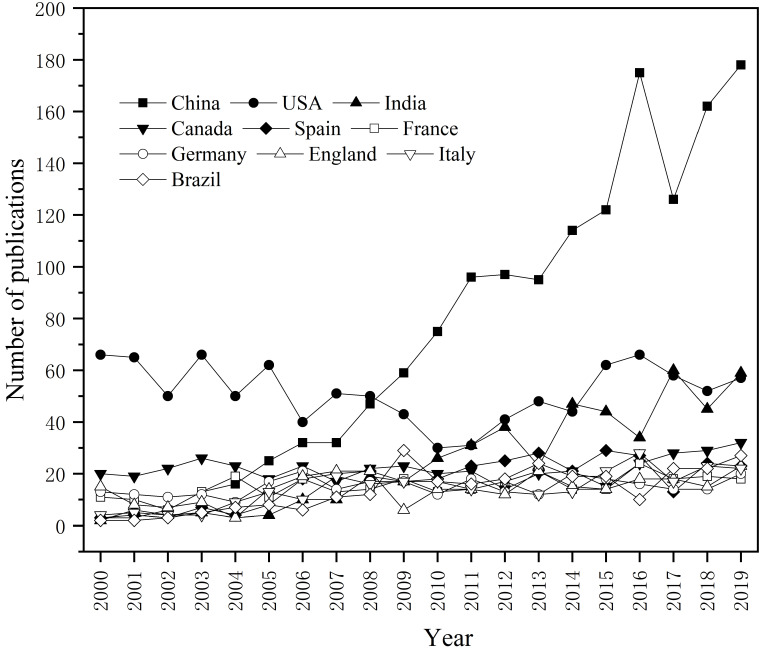
The annual number of each top 10 productive countries on bioremediation of PCS during 2000–2019.

The 7575 records in the WOS database indicate that 90 countries/territories contributed to the bioremediation of PCS publication records. Table 2 shows the top ten countries and territories ranked by the number of total publications produced in each, and also contains other indices, such as total cited frequency and average cited frequency per paper, as well as the country’s *h*-index. The *h*-index was designed as a score to quantify the impact of scientific research. It is defined by the h of total *N_p_* papers having at least h citations each while other (*N_p_-h*) papers have no more than h citations [34,35]. The *h*-index reflects both the quantity (number of publications) and quality (number of citations) of the publications and is therefore widely used to evaluate the scientific research impact of scholars, journals, and countries [36,37]. The top 10 countries were responsible for 68.9% of the total number of publications. The number of publications, citation frequency, and *h*-index of the United States were in the top places, indicating that the United States had a relatively high level of influence in the field. China was the most productive country with 1476 articles, accounting for 19.5% of the total, but the average cited frequency per paper was only 23, which was much lower than that of developed countries, such as Spain, France, Germany, etc. Combined with the annual trend in the number of publications from each top 10 productive country (Figure 2), it was speculated that this might be caused by China’s relatively late start in the field of PCS bioremediation.

Figure 2 shows the top 10 most productive countries with respect to the time-trend analysis during 2000–2019. The research on PCS bioremediation in the United States started early, while the number of articles published from 2000 to 2009 showed a stable state, and the number of articles published increased year by year since 2010. Although China started a little bit late in the field of PCS bioremediation, the number of articles published increased rapidly. In 2009, China surpassed the United States as the country with the largest number of publications. This shows that in recent years, with the rapid development of China’s economy and the acceleration of industrialization, the problem of PCS in China had become increasingly prominent and had gradually received extensive attention. Simultaneously, the remediation technology had also gradually developed from high-cost and irreversible physical and chemical technology to low-cost, no secondary pollution biotechnology and biological combined remediation technology. In addition to China and the United States, the annual number of publications issued by the other eight major countries showed an increasing trend year by year.

**Figure 3:**
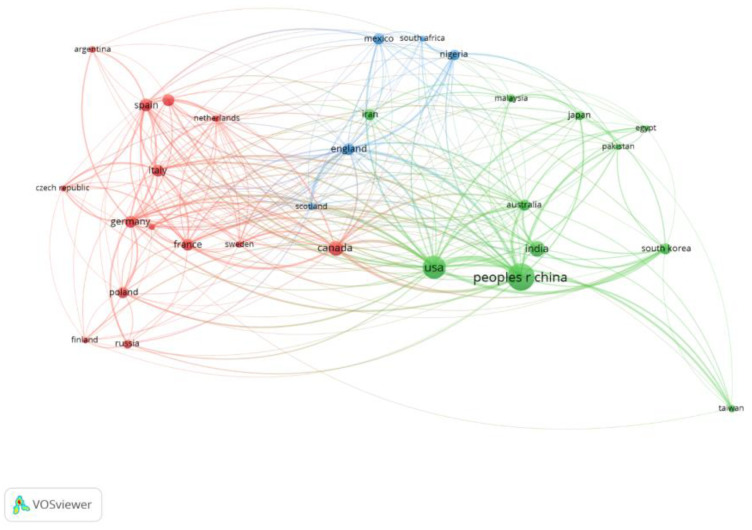
The cooperation network of core countries/territories on bioremediation of PCS during 2000–2019.

For further study, VOSviewer software was used to visualize the cooperative relationships among the top 30 productive countries and regions during 2000–2019, and the results are shown in Figure 3. Each country or region is represented by a circle, and its size depends on the number of publications produced by that particular country. The curve connecting the two circles represents a cooperative relationship between the two linked countries. The thicker the curve, the stronger the collaborations between the two countries. The color of the circle in the visualization networks is determined by the cluster to which the country belongs. The distance between circles implies the degree of cooperation between countries or regions. As can be seen from Figure 3, there were close cooperative relations among all countries. The United States had the most cooperative relations, cooperating with 28 countries or regions, with a total cooperation intensity of 438. China cooperated with 27 countries or regions, mainly the United States, Canada, and England, with a total cooperation intensity of 365. Of all the countries and regions that collaborated, China and the United States had the largest strength of cooperation, which illustrates that the two countries had the closest cooperation.

### 3.3. Publication Distribution of Institutions

**Figure 4:**
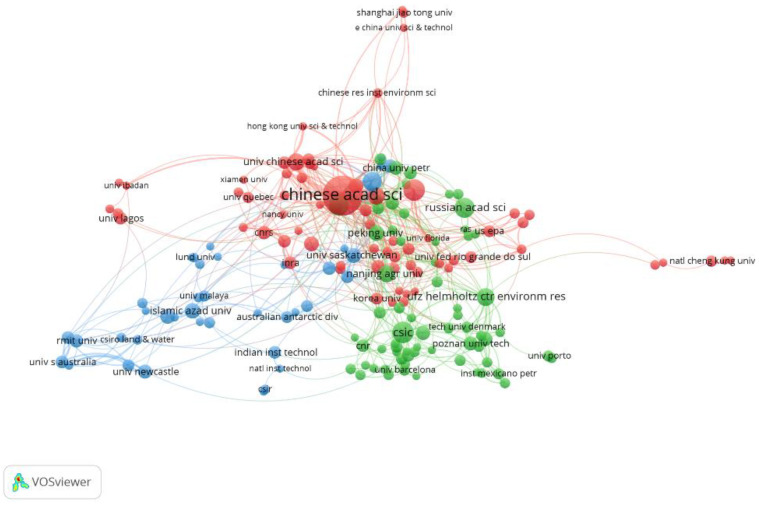
The cooperation network of core institutions on bioremediation of PCS during 2000–2019.

A total of 4070 institutions were involved in the 7575 publications related to PCS bioremediation during 2000–2019. The top 10 most productive institutions in terms of total publication numbers are shown in Table 3. The top 10 institutions originated from six countries—namely, China (4), France (2), India (1), Russia (1), Spain (1), Germany (1)—and accounted for 1329 total publications (17.5%). The United States, Canada, England, Italy, and Brazil belonged to the 10 most productive countries and regions. However, none of these countries’ institutions appeared in the list of the top 10 most productive institutes. The Chinese Academy of Sciences reported the highest number of publications, with 347 articles, which accounted for 4.6% of the total number of citations. The total frequency of citations was also the highest, which was 8994. From the total number of publications, citation frequency, and *h*-index, it can be seen that the Chinese Academy of Sciences has made outstanding contributions in the field of PCS bioremediation. The average citations of articles published by two French institutions, Center National De La Recherche Scientifique and Universite De Lorraine, reached 39.2 and 39.6, respectively, indicating that they made a significant contribution to the field of PCS bioremediation. VOSviewer software was used to visualize the cooperative relationships among the research institutions with more than 15 articles published in the field of PCS bioremediation. The results are shown in Figure 4. There were close and complex cooperative relationships among almost all institutions, and the cooperation network can be divided into three clusters: red, green, and blue. The red cluster is represented by the Chinese Academy of Sciences, primarily consisting of research institutions form China, South Korea, and the United States; the green cluster is chiefly composed of institutions from European countries, such as Spain and Russia; the blue cluster mainly is comprised institutions from Australia, and also includes some institutions from Middle East countries, such as Iran, and Asian countries, such as India. As shown in Figure 4, the Chinese Academy of Sciences was not only the institution with the largest number of papers but also the institution with the largest partnership and cooperation intensity. It cooperated with 52 institutions, with a total cooperation intensity of 220. This shows that the Chinese Academy of Science made an outstanding contribution and had great influence in PCS bioremediation. All research institutions were inclined to cooperate with other domestic institutions. Therefore, strengthening cooperation with overseas institutions is suggested.

### 3.4. Publication Distribution of Journals

The 7575 collected articles related to bioremediation of PCS originated in 868 journals. Table 4 lists the top 10 most active journals concerning the domain of PCS bioremediation. From 2000 to 2019, 2095 articles were published in the top 10 journals in this field, accounting for 27.7% of the total number of articles. The average impact factor in the past five years was 5.536, and the countries in which the journals were published were all developed countries. *Chemosphere* was the most productive journal, with 360 records, accounting for 4.8% of the total number of articles, and its five-year average impact factor was 5.705. It was followed by *Journal of Hazardous Materials* and *International Biodeterioration & Biodegradation* with 307 and 278, respectively. In terms of average citation frequency, the articles published in *Bioresource Technology* had the highest average citation frequency (55.96), which was much higher than that of other journals, followed by *Environmental Science & Technology (47.47)*, which may be related to their high impact factors. The higher the impact factor of a journal, the greater the impact of the journal. *Bioresource Technology* ranked first in the agricultural engineering category, and its five-year average impact factor was 7.27. *Environmental Science & Technology* ranked sixth in the engineering and environmental category, with the highest five-year average impact factor (8.543) among the 10 journals.

### 3.5. The Most Highly Cited Articles

The top 10 highly cited publications in the field of PCS bioremediation from the years 2000 to 2019 were analyzed with parameters such as the total citations and the institutions of origin, and the results were shown in Table 5. Of all the highly cited articles, two were from developing countries (India and China), and the rest were from developed countries. The most highly cited article was entitled “Effects of biochar and greenwaste compost amendments on mobility, bioavailability and toxicity of inorganic and organic contaminants in a multi-element polluted soil” published in *Environmental Pollution* in 2010, with 683 citations [38]. This article studied the effect of applying biochar and greenwaste compost to a multi-element contaminated soils, and the results highlighted the potential of biochar for contaminated land remediation. The second was entitled “Surfactant-enhanced remediation of contaminated soil: a review” published in *Engineering Geology* in 2001, with 644 citations [39]. This article was a review summarizing the indoor studies, field demonstrations as well as large-scale applications of surfactants to remediate contaminated soils, which highly recapitulated and analyzed previous works. It is worth noting that among these top 10 highly cited publications, five articles were reviews. That could mean that this field has authoritative and comprehensive reviews of concepts, characteristics, and influencing factors that will provide the basis for subsequent research. From the analysis of highly cited literature, it can be seen that the technologies that received high attention on PCS bioremediation were mainly microbial in situ remediation techniques, and the main methods adopted were through the degradation effects of indigenous microorganisms in soils or the addition of exogenous hydrocarbon-degrading bacteria, and the main auxiliary means were the addition of amendments such as biochar.

### 3.6. Keyword Analysis

Keywords are highly concise terms that are related to the research content of an article. Consideration of keyword statistics and analyses of the keywords are helpful in identifying research hotspots and research directions in a field, and they are essential in monitoring the development of science and programs [20,30]. Articles with records of author keywords in the field of PCS bioremediation were analyzed. A total of 11,515 keywords were recorded by authors, among which 8592 (74.6%) keywords were used only once, 1324 (11.5%) keywords were used twice, 500 (4.3%) keywords were used three times, and 348 (3%) keywords were used more than 10 times. The large number of once-only author keywords probably indicates a lack of continuity in research and a wide disparity in research focuses [40]. Only small numbers of keywords were used more than three times. This might be because the research in PCS bioremediation was mainly concentrated in a small field. For further study, keywords that were the same or close to the search terms, such as “soil”, “oil”, “petroleum”, “bioremediation”, were removed. Keywords that were meaningless and searched, such as “study” and “its”, were ignored. Keywords that were close to each other, such as “composing” and “compost”, were unified. The top 30 author-generated keywords for the study period are listed in Table 6. Available for high-frequency keyword analysis, crude oil and diesel oil have been the main pollutant sources for the last two decades, with polycyclic aromatic hydrocarbons (PAHs), total petroleum hydrocarbons (TPHs), as well as heavy metals being the main pollutants. It should be noted that the frequency of PAHs is much higher than that of TPHs, which means that more attention has been paid to the bioremediation of PAHs pollution. The main bioremediation techniques were phytoremediation, bioaugmentation, biostimulation, and composting [17]. The main auxiliary means was the addition of biosurfactants (BS), such as rhamnolipids [41]. In addition, the related research on microorganisms, such as the screening of efficient petroleum hydrocarbon-degrading bacteria and the analysis of soil microbial community diversity, is also an important research direction [42,43].

**Figure 5:**
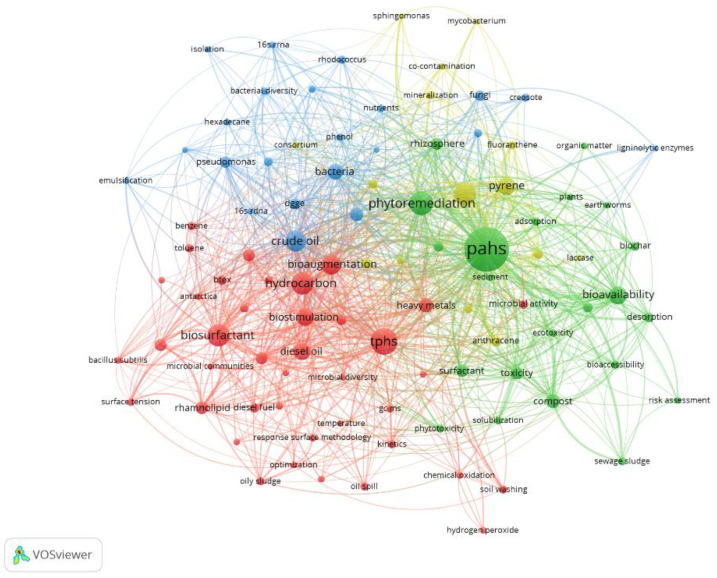
Co-occurrence network of top 100 author keywords on bioremediation of PCS during 2000–2019.

VOSViewer was used to generate a keywords co-occurrence network that shows the connection and weightage of the top 100 most high frequency author keywords, and the results are displayed in Figure 5. A complex and close relationship was formed between the keywords. Each circle represents a keyword. The size of the circle reflects the number of occurrences of a keyword. The connection means a co-occurrence relationship between two keywords, and the color represents the cluster of the keyword—that is, the research topic. As can be seen from Figure 5, keywords were divided into four clusters, cluster 1, cluster 2, cluster 3, and cluster 4 are represented by blue, green, red, and yellow, respectively. The keywords “bacteria”, “fungi”, and “DGGE” indicate that cluster 1 focused on the analysis of microorganisms and the change of microbial diversity, which is more than important for improving the efficiency of bioremediation [44]. From the keywords “phenanthrene”, “pyrene”, and “fluoranthene”, it can be seen that cluster 2 mainly involves the study of the degradation of single pollutants, which is helpful for understanding the degradation characteristics of certain pollutants [45]. Cluster 3 contains keywords related to microbial remediation, such as “biostimulation” and “bioaugmentation”, which are important technologies of microbial remediation [46,47]. In addition, BS strengthening bioremediation of PCS is also an important theme. Cluster 4 focuses on phytoremediation, which can be seen from the keywords such as “phytoremediation”, “plants”, and “rhizosphere”. Cluster 4 also includes the keyword “bioavailability”. Bioavailability is one of the basic principles for judging whether microorganisms are suitable for remediation of contaminants [48]. The study of bioavailability can better evaluate the degradation efficiency of petroleum hydrocarbons by microorganisms and should be the priority research goal in bioremediation [49]. These four clusters reflect the main research content of the current publications on bioremediation of PCS.

### 3.7. Hot Issues

**Figure 6:**
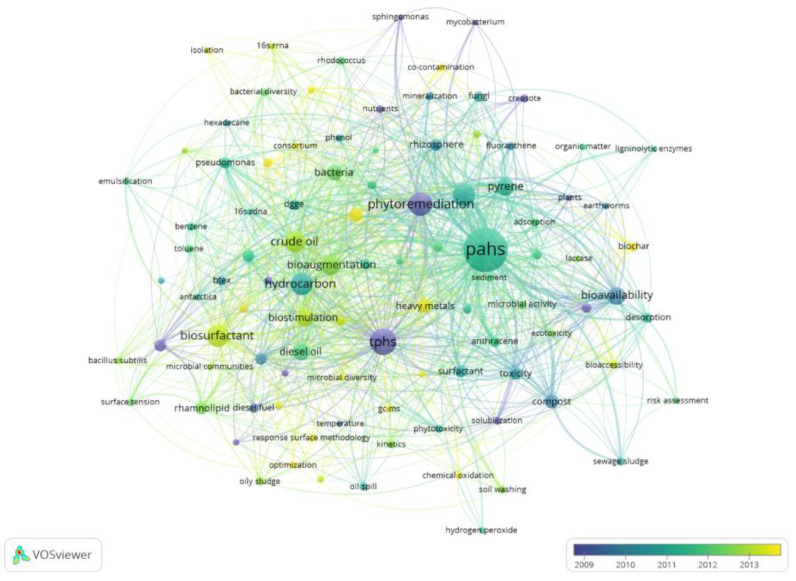
Top 100 author keywords co-occurrence overlap visualization map on bioremediation of PCS during 2000–2019.

The overlay visualization networks reflect the evolution of research content in a certain field and help to understand the research hotspots and development prospects in this field [29]. The same data filtering method was used to generate an overlay visualization network of high-frequency keywords in VOSviewer, as shown in Figure 6. Different colors in the figure represent the average publication year of the literature to which the keyword belongs. The closer the color is to purple, the earlier the keyword appears on average. The closer the color is to yellow, the later the keyword appears on average and the more times it shows in the latest research. As displayed in Figure 6, the average occurrence years of 100 high-frequency keywords were mainly concentrated between 2010 and 2013, and some emerging keywords were scattered among them. Keywords such as “phytoremediation”, “phenanthrene”, and “anthracene” appeared earlier, and keywords such as “microbial community”, “biochar”, “chemical oxidation”, and “heavy metals” appeared later. By analyzing the average time of these high-frequency keywords, combined with the previous highly cited literature analysis and keyword co-occurrence network, this study systematically reflected the evolution of the research topic of PCS bioremediation, and more comprehensively understood the research hotspots and further research directions in this field.

In the early study of petroleum pollution, due to the complex composition of petroleum pollutants, scholars tended to study naphthalene, phenanthrene, anthracene, and other single pollutants at the laboratory level in order to understand the degradation characteristics of certain pollutants. Cerniglia and Yang [50] proved that fungi could oxidize anthracene and phenanthrene to form trans-dihydrodiols. Davies and Evans [51] demonstrated that under aerobic conditions, naphthalene is oxidatively metabolized by soil pseudomonads, undergoing epoxidation and eventual decomposition to carbon dioxide. Kastner and Mahro [52] studied the degradation of naphthalene, phenanthrene, anthracene, fluoranthene, and pyrene in soils and soil/compost mixtures. The results showed that the addition of compost promoted the degradation of other PAHs, such as naphthalene, in soil with low water content.

Phytoremediation has attracted the extensive attention of scholars at home and abroad in the early stage of bioremediation research because of its advantages of safe operation, low cost, sustainability, and environmental friendliness [53,54,55]. Phytoremediation technology refers to the collective term of environmental technology that uses the plant root system (or stem and leaf) to absorb, adsorb, transfer, enrich, degrade, or immobilize contaminants in contaminated soil, water, and the atmosphere [56,57]. It can be divided into phytostabilization, phytostimulation, phytotransformation, phytofiltration, and phytoextraction [58]. The mechanisms of phytoremediation mainly include the plant’s own absorption and metabolism mechanisms of pollutants and the plant’s rhizosphere remediation mechanisms of these two types [59]. Rhizosphere remediation is the primary phytoremediation mechanism for organic pollutants [60]. Siciliano et al. [61] explored the mechanisms by which phytoremediation systems promoted hydrocarbon degradation in soil. The results suggested that phytoremediation systems enhanced the catabolic potential of rhizosphere soils by altering the functional composition of rhizosphere microbial communities. In phytoremediation, screening natural oil-tolerant plants as well as strengthening their growth ability in PCS are critical factors in the success of phytoremediation [62]. Merkl et al. [63] investigated the effects of three legumes (Calopogonium mucunoides, Centrosema brasilianum, Stylosanthes capitata) and three kinds of grass (Brachiaria brizantha, Cyperus aggregatus, Eleusine indica) on the remediation of heavy crude oil contaminated soil. The oil content of soil seeded with grasses was significantly lower compared with the control group. Although phytoremediation is in line with the concept of sustainable development, due to the long growth cycle of plants, slow repair speed, and limited by environmental conditions, it cannot be the most ideal restoration scheme.

With the passage of time, there was more and more research on the application of bioremediation technology to the actual soil pollution [64]. Therefore, the research on petroleum pollutants has changed from a single pollutant to comprehensive research on total petroleum hydrocarbons (TPHs) and polycyclic aromatic hydrocarbons (PAHs). PAHs, which are classified as priority environmental pollutants by the US Environmental Protection Agency, are important components of petroleum hydrocarbons and present carcinogenic, teratogenic, and mutagenic hazards to humans and other organisms [65,66]. Because PAHs are stable in nature, difficult to degrade, and easy to accumulate in soils, PAHs remaining in soils not only seriously contaminate the soil environment but also have potential impact on human health. Therefore, it is necessary to study the remediation of PAHs in soil [67]. Guerin [68] used an ex-situ land treatment process with soil mixing, aeration, and slow-release fertilizer addition for remediation of soils from polycyclic aromatic hydrocarbon pollutants. The result showed that bioremediation significantly degraded low-molecular-weight PAHs by 97% and high-molecular-weight PAHs by 35%.

At the same time, with people’s deeper research on soil microorganisms, microbial remediation technology has gradually become a research hotspot at home and abroad. It refers to the use of the catabolic effect of microorganisms to degrade oil hydrocarbon pollutants in soils as a carbon source, eventually eliminating the pollutants [17]. The essence of microbial remediation is biodegradation and biotransformation. Microbial remediation can be divided into two categories: biostimulation and bioaugmentation [69]. Biostimulation refers to identifying and adjusting certain physical and chemical factors (such as temperature, pH, nutrients, etc.) based on the optimal conditions for indigenous degradation bacteria to improve the abundance and reactivity of indigenous microorganisms, so as to enhance the degradation effect of pollutants [70,71]. Bioaugmentation refers to inoculating exogenous bacteria with degradation function into soil to achieve rapid and efficient removal of pollutants [72,73]. Abdulsalam et al. [74] conducted a study and comparison on biostimulation and bioaugmentation for remediation of soil contaminated with spent motor oil using aerobic fixed bed bioreactors. The results showed that the removal rates of oil by bioaugmentation and biostimulation were 66% and 75%, respectively. Kauppi et al. [75] studied the effects of biostimulation and bioaugmentation on diesel oil contaminated soil in cold regions. The results showed that bioaugmentation is an effective way to improve the efficiency of bioremediation. Suja et al. [71] used a combination of biostimulation and bioaugmentation to remediate crude oil contaminated soil. The results showed that the combination of bioaugmentation with microbial consortium and biostimulation with nutrients was the best treatment method for a contaminated site. Microbial remediation is a widely used remediation method because of its short cycle, easy operation, low cost, and no secondary pollution [76,77]. However, its response to environmental changes is relatively strong, and the degradation efficiency of natural microorganisms is low [78], while inoculated microorganisms have the problem of competition with indigenous microorganisms [79]. Therefore, single microbial remediation is not the best model.

With the development of this field, the trend in research on bioremediation of PCS is expected to continue to mature, especially in the following four main aspects.

#### 3.7.1. Research on the Composite Pollution System of Oil and Heavy Metals

Since petroleum contains heavy metals such as cadmium, lead, and mercury, petroleum hydrocarbon pollution is often accompanied by heavy metal contamination [80,81]. A huge number of studies have pointed out that heavy metal pollution exists in oil fields or industrial soils [82,83]. Vanadium (V) and nickel (Ni) are found in large concentrations in crude oil [84,85], and drilling muds contain large amounts of heavy metals such as lead (Pb), chromium (Cr), zinc (Zn), cadmium (Cd), and copper (Cu) [86]. The composite pollution of petroleum hydrocarbons and heavy metals in soil not only alters soil biosystem structure and adversely affects the stability of biodiversity and soil ecological function, but also accumulates along the food chain in plants, animals, and humans, causing serious threats to human health [13,87]. The interaction between petroleum hydrocarbons and heavy metals will change the form and bioavailability of pollutants, inhibit the activity of degrading bacteria, and make the remediation process more complex [88,89]. At present, there are few studies on the bioremediation of multiple-contaminations of heavy metals and petroleum hydrocarbon. The interaction mechanism of petroleum hydrocarbons and heavy metals in soil and the biodegradation mechanism of petroleum hydrocarbons under heavy metal stress need further study.

#### 3.7.2. Research on the Succession of Soil Microbial Community in the Process of Bioremediation

The degradation of petroleum hydrocarbons is often the result of a community-interacting microbial population [90]. Understanding the changes of soil microbial diversity and activity in the bioremediation process is essential for understanding the behavior and function of the population and ensuring the effectiveness of bioremediation [91]. A large number of studies have used biotechnology to analyze the changes of microbial community in the process of bioremediation. Ros et al. [92] used aeration and organic amendment to remediate semi-arid soil contaminated by oily sludge. The changes of microbial community function and structure after bioremediation were studied by real-time PCR, BIOLOG, and DGGE. It was shown that bioremediation processes led to an increase in soil bacterial abundance, a decrease in microbial diversity, and changes in bacterial community structure and function. Sun et al. [93] used 16S rRNA high-throughput sequencing technology to analyze the microbial community of nine kinds of PCS in Daqing and Changqing oil fields of China. The results showed that many dominant genera in PCS have phylogenetic relationships with known oil degrading species. With the development of molecular biology technology, more and more technologies for monitoring the dynamics of microbial communities will be applied in bioremediation, such as degradative enzyme assays, metagenomic/nucleic acid-based techniques, and phospholipid fatty acid analysis.

#### 3.7.3. Application of BS in Bioremediation

BS is a surface active compound synthesized as metabolic products of different microorganisms [94] that are able to reduce the interfacial tension, surface tension, and the critical micelle concentration and that are able to increase the surface area of hydrophobic pollutants, such as hydrocarbons, and improve their solubility and bioavailability [95], thereby promoting the growth of microorganisms and the degradation of pollutants [96,97]. BS have the characteristics of low toxicity, biodegradability, and specific activity in extreme conditions [98,99]. They have broad application prospects in the bioremediation of PCS. Bezza and Chirwa [3] showed that the degradation rate of PAHs in waste engine oil with BS was as high as 82%, which was more than twice as high as that without BS. Although many studies have proved that BS positively affected the removal of petroleum hydrocarbons at the laboratory level, some field studies have observed that BS have had no significant positive effect and may even have a negative impact on the pollutant treatment process [100,101]. This may be due to the BS deposition on the oil–water interface, which limits the contact between microorganism and substrate, thus inhibiting the biodegradation rate [100]. In the future, more attention should be paid to elucidating the complex relationship between BS, microorganisms, and pollutants.

#### 3.7.4. Application of Biological Combined Remediation Technology

Combined remediation technology organically integrates two or more repair methods, which can fully play to the advantages of each technology and improve remediation efficiency. At present, the biological combined remediation technology mainly includes bio-chemical remediation [102] and inter-organismal (including plants, animals, microorganisms) remediation [103,104]. Among them, phyto–microbial remediation and chemical oxidation–microbial remediation are currently widely used biological combined remediation technologies to restore PCS [105]. Phyto–microbial remediation technology utilizes the synergy between plants and microorganisms to immobilize, absorb and degrade pollutants [103]. On the one hand, plant roots provide a living place for microorganisms [44], and the chemical substances secreted by them improve the bioavailability of petroleum pollutants, which is conducive to microbial metabolism and decomposition [106,107]. On the other hand, microorganisms increase the biomass of plant roots, and their co-metabolism also improves the utilization efficiency of petroleum pollutants [102,108]. Xun et al. [109] studied the effects of plant growth-promoting bacteria (PGPR) and arbuscular mycorrhizal fungi (AMF) on the remediation of petroleum-contaminated saline alkali soil by oat plants. The results showed that the combination of PGPR and AMF made the plants more tolerant to petroleum hydrocarbons pollutants. Chemical oxidation combined with microbial remediation technology takes chemical oxidation as the pretreatment of bioremediation, which can improve the water solubility of petroleum hydrocarbons and transform the refractory macromolecular organic pollutants into small molecular substances [110]. Gong [111] used biostimulation and improved Fenton oxidation to decontaminate crude-oil-polluted soil. The results showed that the TPH content of the combined treatment decreased by 88.9%, while that of the biological treatment alone reduced by 55.1%. Biological combined remediation is the research hotspot in the field of PCS remediation. However, due to the differences between laboratory and remediation site environments, the specific effect of biological combined remediation on degradation of petroleum pollutants still needs to be verified by field research.

In general, based on keyword visualization analysis in the field of bioremediation of PCS, new research focuses on the compound pollution system of oil and heavy metals and how to improve the efficiency of bioremediation. As time goes on, the related research may be more abundant and in-depth.

## 4. Conclusions

In this study, bibliometric analysis was conducted based on 7575 related articles on the bioremediation of PCS from 2000 to 2019. The following conclusions were drawn from this study:

(1) A steady increase was observed in publication output, with extensive international collaboration in the past 20 years. The growth rate in the number of articles published from 2000 to 2019 was 21.7%.
(2) China was the country with the highest number of articles (1476), and the United States had the highest *h*-index (86). The Chinese Academy of Sciences was the institution with the largest number of papers (347) and cooperative relations (52).
(3) *Chemosphere* was the most productive journal, with 360 records. The most highly cited article was on applying biochar and compost in soil remediation and was published by the Liverpool John Moores University in *Environmental Pollution* in 2010, with 683 citations.
(4) According to the analysis of high-frequency keywords, the research on PCS bioremediation was basically steady. Phytoremediation and microbial remediation were the main remediation methods.
(5) More study on the following research areas can be conducted: clarifying the biodegradation mechanism of TPHs under heavy metal stress; monitoring microbial community dynamics during bioremediation; expounding the relationship between BS, microorganisms, and pollutants; and carrying out field studies on biological combined remediation of PCS.

Despite the contributions of the study, we must mention that it still has some limitations. We only used a single database instead of different sources to retrieve the information. Publications outside the WOS Core Collection database and citations outside the WOS registered journals were neither included nor analyzed, which may have excluded some influential articles. In addition, some of the documents we retrieved were weakly related to the bioremediation of PCS. Manual screening is difficult, time-consuming, and highly subjective. Therefore, further research should use multiple databases to retrieve publications and should use text mining tools to filter results, which would help to improve the accuracy and scientificity of analysis.

Overall, these results generated by bibliometric analysis reveal the global research trends in bioremediation of PCS. Thus, this research helps researchers understand the development trends and research themes in bioremediation of PCS and provides some guidance for future research.

## Figures and Tables

**Table 1:** Characteristics of annual publications on bioremediation of PCS during 2000–2019.

PY	TP	AU	AU/TP	PG	PG/TP	NR	NR/TP
2000	191	592	3.1	1801	9.4	5862	30.7
2001	195	578	3.0	1813	9.3	5556	28.5
2002	168	544	3.2	1572	9.4	4858	28.9
2003	221	737	3.3	2139	9.7	6873	31.1
2004	215	699	3.3	2010	9.3	7252	33.7
2005	252	867	3.4	2334	9.3	8420	33.4
2006	286	988	3.5	2626	9.2	9719	34.0
2007	309	1057	3.4	2681	8.7	10,403	33.7
2008	372	1312	3.5	3186	8.6	12,955	34.8
2009	367	1402	3.8	3087	8.4	13,011	35.5
2010	366	1386	3.8	3279	9.0	14,287	39.0
2011	390	1527	3.9	3360	8.6	15,259	39.1
2012	441	1688	3.8	3878	8.8	17,440	39.5
2013	427	1651	3.9	4067	9.5	18,254	42.7
2014	459	1856	4.0	4287	9.3	19,012	41.4
2015	537	2179	4.1	5394	10.0	23,173	43.2
2016	597	2435	4.1	5972	10.0	27,340	45.8
2017	542	2290	4.2	5543	10.2	25,725	47.5
2018	616	2611	4.2	6354	10.3	29,952	48.6
2019	624	2815	4.5	6609	10.6	31,681	50.8
Total	7575	29,214	3.7	71,992	9.4	307,032	38.1

TP: number of publications; AU: number of authors; PG: page count; NR: cited reference count; and AU/TP, PG/TP and NR/TP: average number of authors, pages, references per article.

**Table 2:** Top 10 countries in volume of publications on bioremediation of PCS during 2000–2019.

No.	Country	TP	R/%	NC	NC/TP	*h*-Index
1	China	1476	19.485	33,997	23	78
2	USA	1032	13.624	32,212	31.2	86
3	India	487	6.429	11,685	24	53
4	Canada	437	5.769	13,104	30	60
5	Spain	336	4.436	11,942	35.5	56
6	France	305	4.026	10,656	34.9	51
7	Germany	300	3.96	9677	32.3	49
8	UK	287	3.789	9299	32.4	52
9	Italy	285	3.762	7236	25.4	46
10	Brazil	277	3.657	6176	22.3	39

TP: the number of total publications; R (%): the ratio of the number of one country’s publications to the total number of publications during 2000–2019; NC: the number of citations; NC/TP: average number of citations per article.

**Table 3:** Top 10 institutions in volume of publications on bioremediation of PCS during 2000–2019.

No.	Institutions	TP	R/%	NC	NC/TP	*h*-Index
1	Chinese Academy of Sciences	347	4.581	8994	25.9	47
2	Center National De La Recherche Scientifique	163	2.152	6390	39.2	40
3	Helmholtz Association	132	1.743	3701	28	35
4	Consejo Superior De Investigaciones Cientificas	126	1.663	4698	37.3	43
5	Russian Academy of Sciences	111	1.465	1266	11.4	19
6	Zhejiang University	98	1.294	3316	33.8	31
7	University of Chinese Academy of Sciences	94	1.241	2454	26.1	28
8	Council of Scientific Industrial Research	87	1.149	2224	25.6	27
9	Tsinghua University	86	1.135	2054	23.9	27
10	Universite De Lorraine	85	1.122	3369	39.6	33

TP: the number of total publications; R (%): the ratio of the number of one country’s publications to the total number of publications during 2000–2019; NC: the number of citations; NC/TP: average number of citations per article.

**Table 4:** Top 10 journals of publications on bioremediation of PCS during 2000–2019.

Rank	Journals	TP	R/%	NC	NC/TP	IF (5 Years)	Country
1	*Chemosphere*	360	4.752	14,828	41.19	5.705	UK
2	*Journal of Hazardous Materials*	307	4.053	12,773	41.61	8.512	Netherlands
3	*International Biodeterioration & Biodegradation*	278	3.67	8607	30.96	4.046	UK
4	*Environmental Science and Pollution Research*	224	2.957	3792	16.93	3.306	Germany
5	*Environmental Science & Technology*	177	2.337	8402	47.47	8.543	USA
6	*Water Air and Soil Pollution*	164	2.165	2748	16.76	2.041	Netherlands
7	*Science of The Total Environment*	162	2.139	4120	25.43	6.419	Netherlands
8	*Environmental Pollution*	157	2.073	7252	46.19	6.939	USA
9	*Bioresource Technology*	139	1.835	7779	55.96	7.27	Netherlands
10	*Biodegradation*	127	1.677	3625	28.54	2.575	Netherlands

TP: the number of total publications; R (%): the ratio of the number of one country’s publications to the total number of publications during 2000–2019; NC: the number of citations; NC/TP: average number of citations per article; IF: impact factor.

**Table 5:** Top 10 highly cited papers in citation frequency on bioremediation of PCS during 2000–2019.

Rank	Title	Country of Corresponding Author	Publication Year	Journal	NC
1	Effects of biochar and greenwaste compost amendments on mobility, bioavailability and toxicity of inorganic and organic contaminants in a multi-element polluted soil	England	2010	*Environmental Pollution*	683
2	Surfactant-enhanced remediation of contaminated soil: a review	Canada	2001	*Engineering Geology*	680
3	An overview on olive mill wastes and their valorisation methods	Spain	2006	*Waste Management*	451
4	Comparative bioremediation of soils contaminated with diesel oil by natural attenuation, biostimulation and bioaugmentation	USA	2005	*Bioresource Technology*	405
5	Bioavailability of hydrophobic organic contaminants in soils: fundamental concepts and techniques for analysis	England	2003	*European Journal of Soil Science*	386
6	Degradation and mineralization of high-molecular-weight polycyclic aromatic hydrocarbons by defined fungal-bacterial cocultures	Australia	2000	*Applied and Environmental Microbiology*	385
7	Crude petroleum-oil biodegradation efficiency of Bacillus subtilis and Pseudomonas aeruginosa strains isolated from a petroleum-oil contaminated soil from North-East India	India	2007	*Bioresource Technology*	362
8	Plant uptake, accumulation and translocation of phenanthrene and pyrene in soils	China	2004	*Chemosphere*	348
9	Two complementary sides of bioavailability: Accessibility and chemical activity of organic contaminants in sediments and soils	Denmark	2006	*Environmental Toxicology and Chemistry*	343
10	Bioremediation of heavy metals from soil and aquatic environment: An overview of principles and criteria of fundamental processes	South Korea	2015	*Sustainability*	342

NC: the number of citations.

**Table 6:** Top 30 author keywords of published papers on bioremediation of PCS during 2000–2019.

Keywords	Frequency	Keywords	Frequency	Keywords	Frequency
Polycyclic aromatic hydrocarbons	1199	Diesel oil	182	Rhizosphere	85
Total petroleum hydrocarbons	449	Biostimulation	179	Toxicity	85
Phytoremediation	342	Bacteria	152	Pseudomonas aeruginosa	84
Hydrocarbons	314	Compost	121	Pseudomonas	81
Biosurfactant	307	Heavy metals	119	Anthracene	72
Phenanthrene	296	Microbial community	116	Fungi	72
Crude oil	259	Rhamnolipid	99	DGGE	69
Bioaugmentation	237	Natural attenuation	88	Naphthalene	69
Pyrene	204	Surfactant	88	Sorption	61
Bioavailability	199	Groundwater	87	Microorganisms	60

## Data Availability

Data is contained within the article.

## Abbreviations

PCS, petroleum contaminated soils; TPHs, total petroleum hydrocarbons; PAHs, polycyclic aromatic hydrocarbons; WOS, Web of Science; TP, number of publications; AU, number of authors; PG, page count; NR, cited reference count; NC: the number of citations; AU/TP, PG/TP, NC/TP, and NR/TP: average number of authors, pages, citations, and references per article. DGGE, denaturing gradient gel electrophoresis; PCR, polymerase chain reaction; BS, biosurfactants; PGPR, plant growth-promoting bacteria; AMF, arbuscular mycorrhizal fungi.
